# Deciphering the BAR code of membrane modulators

**DOI:** 10.1007/s00018-017-2478-0

**Published:** 2017-02-27

**Authors:** Ulrich Salzer, Julius Kostan, Kristina Djinović-Carugo

**Affiliations:** 1 Max F. Perutz Laboratories, Department of Medical Biochemistry, Medical University of Vienna, Dr. Bohr-Gasse 9, 1030 Vienna, Austria; 2 Max F. Perutz Laboratories, Department of Structural and Computational Biology, University of Vienna, Campus Vienna Biocenter 5, 1030 Vienna, Austria; 3 Department of Biochemistry, Faculty of Chemistry and Chemical Technology, University of Ljubljana, Večna pot 119, 1000 Ljubljana, Slovenia

**Keywords:** N-BAR domain, F-BAR domain, I-BAR domain, lipid binding, Membrane remodelling, Membrane curvature

## Abstract

The BAR domain is the eponymous domain of the “BAR-domain protein superfamily”, a large and diverse set of mostly multi-domain proteins that play eminent roles at the membrane cytoskeleton interface. BAR domain homodimers are the functional units that peripherally associate with lipid membranes and are involved in membrane sculpting activities. Differences in their intrinsic curvatures and lipid-binding properties account for a large variety in membrane modulating properties. Membrane activities of BAR domains are further modified and regulated by intramolecular or inter-subunit domains, by intermolecular protein interactions, and by posttranslational modifications. Rather than providing detailed cell biological information on single members of this superfamily, this review focuses on biochemical, biophysical, and structural aspects and on recent findings that paradigmatically promote our understanding of processes driven and modulated by BAR domains.

## Introduction

**Fig. 1:**
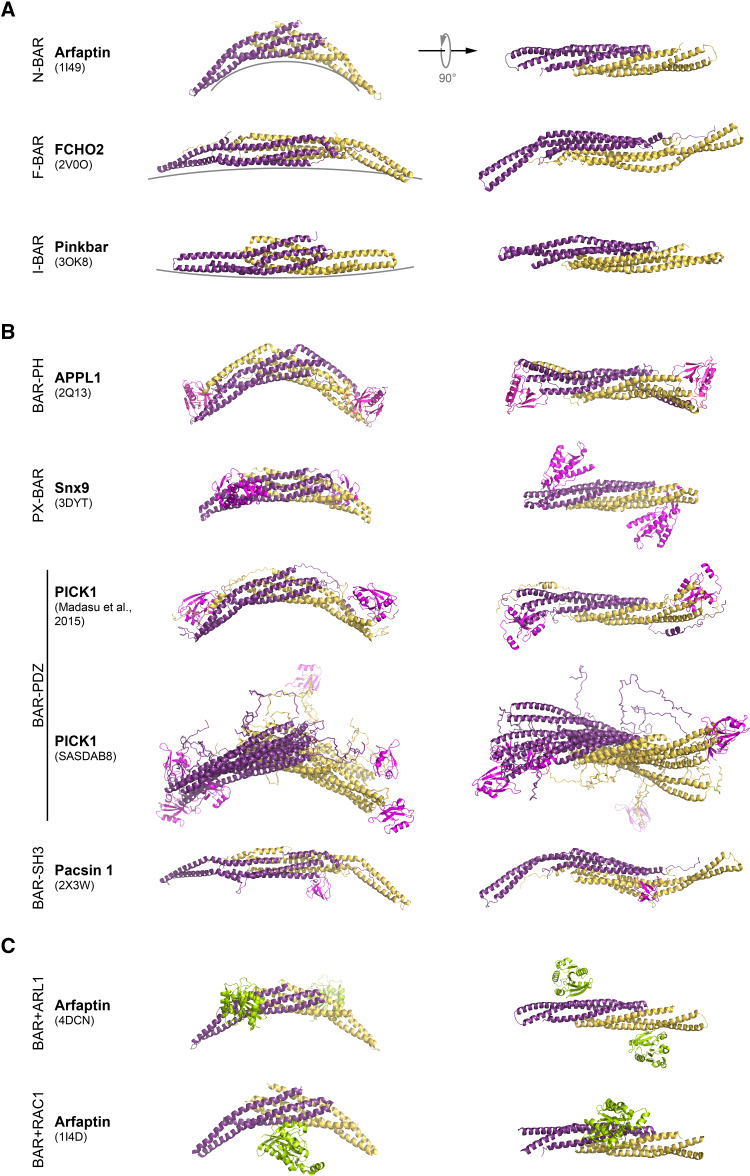
Structure of selected BAR domain dimers. The BAR domain dimers form an elongated structure with a core bundle of six α-helices generated by antiparallel dimerisation of two BAR domain monomers. 3D structures of BAR domain dimers are shown as a ribbon. Monomers are depicted in different colors (*yellow* and *dark magenta*). *Side view* of the each BAR dimer is shown on *left*, while *top view* is on *right*. **a** Examples of BAR domain dimers representing N-BAR, F-BAR, and I-BAR domain fold. Different degrees of curvature adopted by each class of BAR domain dimers are depicted by *grey lines*. **b** Structures of BAR domain dimers from different subfamilies with their accessory domains (PH, PX, PDZ, and SH3) shown in *magenta*. Note, for PICK1, that two SAXS analysis derived models are shown. In PICK1 model (SASDAB8), the PDZ domains are far apart and flexible with respect to the BAR domain. Here, overlay of three generated models is shown. In the PICK1 model of Madasu et al. [78], the position of the PDZ domain was found to be well constrained, and packed against BAR domain. **c** Structure of the Arfaptin-2 BAR domain dimer in complex with Arl1 GTPase, and Rac1-GDP, both shown in *green*

Identification of sequence homology between the N-terminal regions of the Bin, the amphiphysin, and the yeast Rvs proteins led to the recognition of a novel protein domain which was named “BAR domain” as an acronym composed of the first letters of these proteins [1]. This domain was found in a large set of proteins which were classified as BAR domain proteins, later also termed N-BAR domain proteins, because several members of this protein family have an amphipathic helix at the N-terminus of the BAR domain [2]. The structure of the amphiphysin BAR domain laid the basis for a mechanistic understanding of membrane deformation by this protein and of N-BAR domain proteins, in general: the BAR-domain homodimer displays a crescent shape that binds to the membrane bilayer with its concave side. In addition, an N-terminal amphipathic helix is thought to insert into the membrane like a “wedge”, thereby inducing membrane buckling [2]. Soon thereafter, the relationship on the sequence level between N-BAR domains and a protein domain that consisted of an N-terminal FCH (Fes/CIP4 homology) and a coiled-coil (CC) domain was found and the term “F-BAR domain” was coined [3]. The alternative term “extended FC (EFC) domain” stressing the connection between the FCH and the CC region [4] is less used than “F-BAR domain”, because “BAR” is nowadays well associated with the general notion of membrane modeling. Structural analyses of the IRSp53 protein identified the Rac-binding (RCB) domain/IRSp53-MIM homology domain (IMD) as a member of yet another type of BAR domains [5, 6]. Here, in contrast to the crescent-shaped dimer of the BAR-domain, the dimer of the RCB domain of IRSp53 revealed a “zeppelin-like shape”. Similar to BAR domains, this RCB/IMD domain which was known to bundle actin filaments and bind Rac, displayed membrane deforming activity. Since RCB/IMD induces a membrane curvature opposite that of BAR domains and deforms membranes by binding to the interior of the tubules, this domain was renamed accordingly as “inverse-BAR” or I-BAR domain [7, 8] (Fig. 1a).

**Fig. 2:**
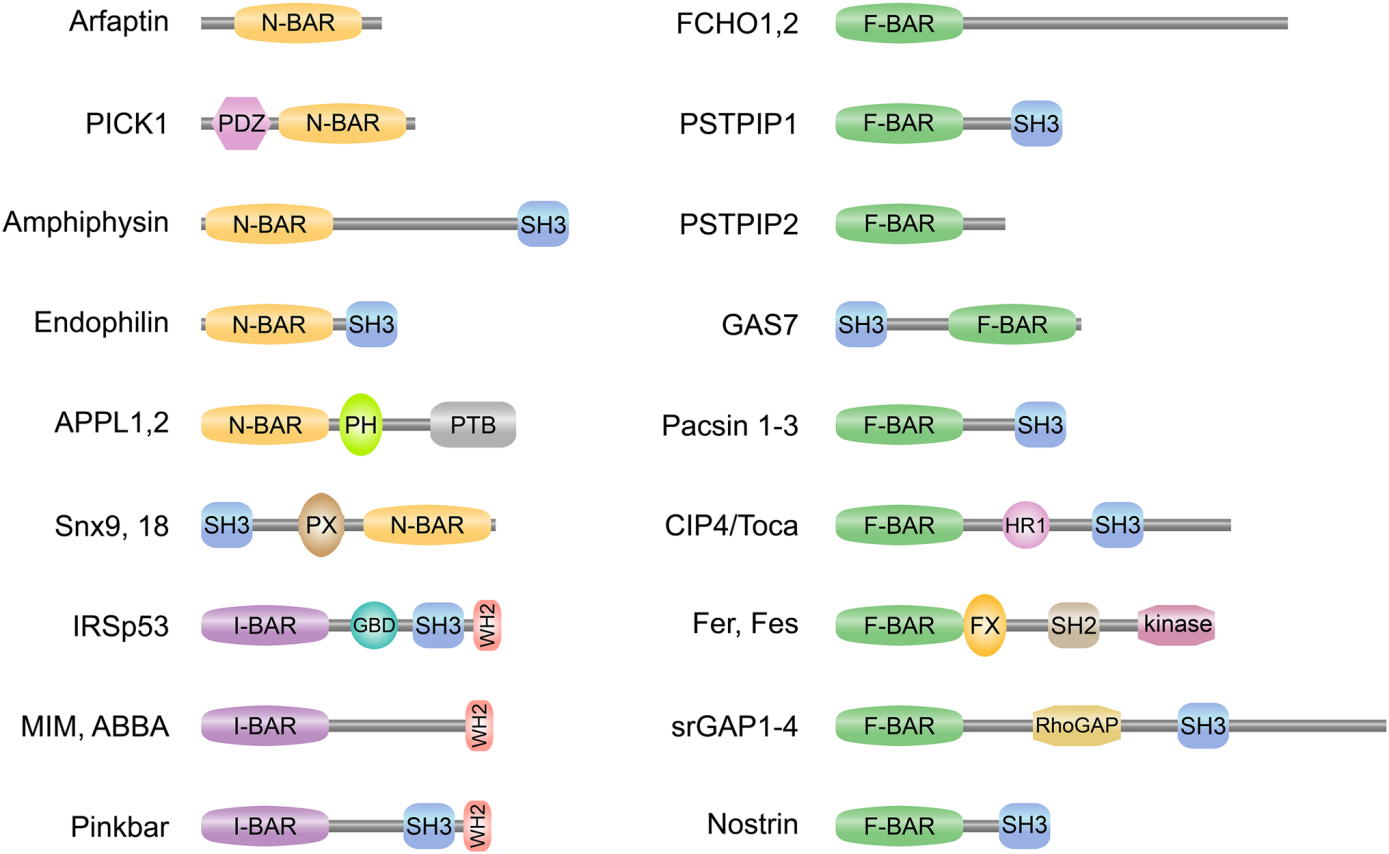
Schematic domain representation of selected BAR domain proteins. Selected members of the N-BAR (*BAR* with an N-terminal amphipathic helix) and I-BAR (inverse-*BAR*) domain family on left and F-BAR (Fes/CIP4 homology-*BAR*) domain family on right are depicted. Most BAR domain proteins contain one or several additional domains with lipid-binding, protein-binding, and/or enzymatic activities. PDZ (PSD95/Dlg1/ZO-1) domain mediates protein–protein interactions by binding to the C-terminus of other specific proteins. SH3 (Src homology 3) domain confers binding to poly-proline motifs of target proteins, like N-WASP or dynamin. The phosphoinositides-binding PX (phox homology) and PH (pleckstrin homology) domains modulate membrane-binding specificities of different subsets of N-BAR domain proteins. PTB (phosphotyrosine-binding) domain binds to phosphotyrosine. GBD (GTPase**-**binding domain) is required for binding to Rho small GTPases. WH2 (Wiskott-Aldrich syndrome homology 2) domain binds to actin monomers and can facilitate the assembly of actin monomers into actin filaments. HR1 (protein kinase C-related kinase homology region 1) binds the small G protein Rho. FX (F-*BAR* extension) domain in Fer was shown to bind phosphatidic acid. SH2 (Src homology 2) domain allows binding to phosphorylated tyrosine residues on other proteins. RhoGAP (Rho GTPase activating protein) domain modulates the activity of Rho. Fer and Fes possess a tyrosine kinase domain

Most BAR domain proteins contain one or several additional domains with lipid-binding, protein-binding and/or enzymatic activities (Figs. 1b, 2). The most common domain combined with N-, F-, and I-BAR domains is the Src Homology 3 domain (SH3) which confers binding to poly-proline motifs of target proteins, like the cytoskeletal organizer N-WASP or the membrane vesicle scissor dynamin [9]. The phosphoinositide-binding phox homology (PX) and pleckstrin homology (PH) domains are present in different subsets of N-BAR domain proteins, thereby modulating membrane-binding specificities of these proteins. A Rho GTPase activating protein (RhoGAP) domain is found in the N-BAR domain proteins nadrin and oligophrenin and the F-BAR domain proteins srGAP1-4 (Fig. 2) and HMHA1. N-BAR domain proteins ASAP1 and centaurin contain an ArfGAP domain and the tuba protein contains a Rho guanine-nucleotide exchange factor domain (RhoGEF). This indicates a close linkage between BAR-domain proteins and small GTPases which are known as master regulators of the actin cytoskeleton. The combination of BAR domains with additional functional domains within the same polypeptide constitutes the functional diversity of the members of this superfamily. BAR domain proteins are key players in processes like clathrin-dependent [10] and clathrin-independent [11] endocytosis, caveolae formation [12, 13], intracellular vesicle formation [14], cell migration [15, 16], and cytokinesis [17] to name only a few. The recognition of the full molecular significance of BAR domain proteins is only slowly emerging. Overviews of our current knowledge on the cell biological function and the (patho)-physiological impact of BAR domain proteins are given in various excellent reviews [18–24].

In this review, we focus on the association of the BAR-domain dimer with intramolecular or intradimer domains as well as on its ligand-binding characteristics. Rather than giving a broad overview on the functional diversity of BAR domain proteins, we here specifically present those studies that further our mechanistic understanding of processes driven/modulated by BAR domain dimers.

## Interaction with membranes

### Lipid-binding specificities

The excess of positively charged residues at the concave side of the crescent-shaped dimer is a hallmark of N- and F-BAR domain proteins and is suggestive for their preferential binding to membrane regions rich in anionic phospholipids [25, 26]. Instead, in I-BAR domain proteins, positively charged residues are accumulated at the convex side of the dimer [6]. Membrane binding of BAR-domain proteins was most thoroughly studied by liposome-binding assays as judged by co-sedimentation and even more specific by co-flotation in a density gradient upon applying centrifugal force. Liposomes were either prepared from membrane lipid extracts or from defined lipid mixtures to assess the lipid-binding specificities of BAR-domain protein. The heterogeneity in the details of the applied methods, however, often impedes the comparability of data from different studies. Moreover, as outlined by Carvalho et al. [27], limited stability of liposomes regarding their lipid composition has to be regarded as a general caveat for the evaluation of studies where these parameters are not tightly controlled.

Using liposomes composed of 80% phosphatidylcholine (PC) and 20% phosphatidylethanolamine (PE) as non-binding control, it was found that replacement of 10% PC by the negatively charged phosphatidylserine (PS) greatly enhanced binding of the F-BAR domain proteins FBP17 and pacsin (syndapin) [3]. Liposome-binding was absent when the PS content was below 5% and saturated when over 10%. A similar replacement by phosphatidic acid (PA) or various species of phosphoinositides only modestly enhanced binding of these proteins compared to control. However, most phosphoinositide species, at a relative fraction of 10%, greatly enhanced binding of FBP17 only when the lipid composition contained additional 5% PS [3]. Similarly, strong binding of pacsin-1 and pacsin-2 to phosphatidyl-inositol-(4,5)-bisphosphate (PIP2) containing lipsomes was dependent on the presence of PS in the lipid mixture [28]. Tsujita et al. showed PIP2-dependent increase in liposome-binding for the CIP4, Fer, and PSTPIP F-BAR domains [4]. Thus, PS or a combination of phosphoinositides and PS is required for membrane binding of these F-BAR domain proteins.

Comparing the requirements of membrane association within the srGAP subfamily of F-BAR proteins, Coutinho-Budd et al. found that srGAP2 and srGAP3 differently depend on PIP2 for membrane association [29]. In contrast to srGAP3, srGAP2 remains largely membrane associated upon temporal cellular PIP2 depletion. Despite of a high degree of similarity between these two proteins, this differential behavior is likely due to altered lipid-binding specificities. The srGAP2 protein apparently has a broader spectrum of affinities to negatively charged membrane lipids and thus withstands conditions of a selective loss of PIP2 in the membranes. These data impressively show the differential influence of membrane lipid composition on the subcellular localization of BAR domain proteins and indicate a general regulatory impact of lipid metabolism on these proteins.

Studying the two F-BAR proteins CIP4 and nostrin that cooperate in the regulation of epithelial morphogenesis, Zobel et al. [30] found that nostrin had the typical PS dependent liposome-binding characteristic of F-BAR domain proteins, whereas CIP4, astonishingly and in contrast to earlier results [4], even bound to liposomes solely composed of 80% PC and 20% PE, indicating that the presence of negatively charged lipids is not a strict requirement for CIP4-membrane binding. The difference in their lipid-binding specificities and membrane tubulating activities is likely the basis for the cooperative regulatory action of CIP4 and nostrin at the endosomal membrane system [30].

A recent study on three yeast F-BAR domain proteins, namely Rgd1p, Hof1p, and Bzz1p, similarly revealed unexpected differences in their lipid-binding specificities [31]. While Rgd1p liposome-binding was greatly enhanced in the presence of PIP_2_, Hof1p, and Bzz1p binding was indifferent or even negatively affected by PIP2, respectively. Moreover, PS-containing and pure PC liposomes were equally efficient in binding Hof1p and Bzz1p proteins, suggesting that negatively charged lipids are not essential for membrane association (at least under these experimental conditions). In agreement with PIP2-enhanced binding to liposomes, structural analysis of the Rgd1p F-BAR domain identified a cluster of five positively charged residues at the concave side, coordinating an inositol-hexa-phosphate molecule [31]. This cluster was absent in the structure of the Hof1p BAR domain. Mutational analyses confirmed the importance of this cluster for the PIP2-binding specificity of Rgd1. This cluster is (partially) conserved in a subset of mammalian F-BAR domain proteins, e.g., in FBP17 and CIP4. However, experimental data indicate that these proteins have less selectivity for phosphoinositides and/or interact with different species of membrane lipids. Thus, interactions of BAR domain proteins with lipids are complex and the code that determines its specificities is far from being understood yet.

The sorting nexin subfamily is defined by a phox homology (PX) domain, a phospholipid-binding module, C-terminal to the N-BAR domains (Fig. 1). SNX9 is involved in clathrin-mediated endocytosis and has been shown to stimulate N-WASP-mediated activation of the Arp2/3 complex and promote F-actin branching [32, 33]. The activation of N-WASP by SNX9 is enhanced by PIP2 and higher order oligomer formation of SNX9 [32]. In liposome assays, SNX9 did not efficiently bind to liposomes composed of PC (70%), PE (15%) and PS (15%) but binding increased considerably upon addition of PIP2 (7.5%) [34]. SNX9 turned out to have a broad binding specificity for different phosphoinositide species, which all synergistically enhance the activation of N-WASP by SNX9. This is in line with the notion that SNX9 is functionally active at various subcellular membranes with differential composition of phosphoinositides. Moreover, mutational analyses revealed that the PX and the N-BAR domains both contribute to efficient lipid-binding and are both required for localization of SNX9 to clathrin-coated pits.

The I-BAR domains of the MIM and IRSp53 proteins are shown to specifically bind to membranes, depending on the presence of the phosphoinositides PI(4,5)P2 and to a lesser extent on PI(3,4)P2; PIP3, PIP, PI, or PS do not significantly increase their affinity to liposomes [8]. The binding to PIP2 is conferred by relatively large clusters of positively charged residues mapping to the distal ends of the I-BAR domain. Mutants defective in PIP2 binding also showed a significant loss in filopodia-inducing activity. Using giant unilamellar vesicles (GUVs) containing differentially labeled PC and PIP2, Saarikangas et al. found that I-BAR domains of IRSp53, MIM, and ABBA induce visible clusters of PIP2 on GUVs [7]. The N-BAR domain of amphiphysin clustered PIP2 with significantly less efficiency. Interestingly, amphiphysin clustered PIP2 and PS with equal efficiency, whereas clustering by the I-BAR domain was specific for PIP2 [7]. Furthermore, the yeast F-BAR domain proteins Syp1, Bzz1, and Rvs161/167 were also found to cluster phosphoinositides with an efficiency of PI(3,4,5)P3 > PI(4,5)P2 > PI3P, indicating that clustering is promoted by electrostatic attraction [35]. In this study, Zhao et al. used FRAP to investigate the lipid dynamics in these clusters and tubular regions induced by these F-BAR domain proteins and found an almost complete lack of lateral diffusion of PIP2 at these sites [35]. They further studied the effect on lipid dynamics of Lsp1, a yeast BAR-domain protein that is involved in the formation and stabilization of eisosomal membrane invaginations. Lsp1 similarly formed stable scaffolds at the membrane that inhibited lateral diffusion of PIP2 and generally decreased membrane fluidity, thereby indicating that it interacts with the acyl-chains of membrane lipids. Thus, BAR-domain proteins—by interacting with membrane lipids—are likely to be involved in microdomain formation at cellular membranes. One biochemical feature of such microdomains—also known as “lipid rafts” [36]—is their insolubility in non-ionic detergents like Triton X-100 (TX-100). Interestingly, the F-BAR domain protein srGAP1, in contrast to the related srGAP2 protein, was implicated to be a lipid raft-associated protein due to its TX-100 insolubility [29].

In an elegant study, Picas et al. investigated factors that confer the BIN1/M-Amphiphysin2-dependent recruitment of dynamin, a process crucial for T-tubule formation in muscle cells [37]. They found that the N-BAR domain of BIN1 clustered PIP2 (and to a lesser extend other phosphoinositides) both in flat membrane sheets in vitro as well as in membrane tubules in cellula upon over-expression and showed that these PIP2 clusters strongly enhanced the kinetics of dynamin recruitment. Molecular dynamics simulations indicated that PIP2 was not strictly sequestered by but reversibly associated with the N-BAR domain of BIN1, thereby being still available for interaction with downstream partners that also contain phosphoinosite binding motifs, like dynamin [37]. Studies like this will be necessary to further evaluate the contribution of the divers lipid-binding properties of BAR-domain proteins to their specific cellular functions.

### Membrane bending, tubulation, and vesiculation

#### N-BAR domain

The membrane tubulation activity of N-BAR domain proteins was first discovered by Takei et al. when using amphiphysin-1 as a control for dynamin-1 in a liposome tubulation assay [9]. Elucidation of the amphiphysin BAR-domain structure as a crescent-shaped dimer laid the basis for the concept that BAR domains are, on one hand, sensors of membrane curvature and, on the other hand, act as a mold to induce local membrane bending [2] (Fig. 1a). The unstructured N-terminus, giving the name to this class of BAR domains, was found to form an amphipathic helix upon lipid-binding and thereby increase the affinity of the BAR domain for membrane association [38]. Three types of curved membrane structures were introduced in liposomes depending on the concentration of the N-BAR domain: small buds at low, elongated tubules at intermediate, and vesicles at high concentrations. A BAR-domain mutant lacking the N-terminal amphipathic helix still showed tubulation activity, yet only at higher concentrations [2]. Positively charged residues at the concave lipid-binding side of the N-BAR domain are involved both in membrane binding and tubule formation.

Membrane bending and tubulation were further extensively studied for the N-BAR domain of endophilin A1 [38, 39], a protein involved in generating endocytic necks and vesicles during synaptic endocytosis. Similar to amphiphysin, the N-terminal amphipathic helix (also termed H_0_ helix) increased the affinity of the BAR domain for membrane lipids and the crescent-shaped dimeric BAR domain itself was critical for membrane tubulation [38]. A mutant with increased flexibility of the arms of the N-BAR dimer lost the ability to tubulate liposomes, indicating that this activity was dependent on the structural rigidity of the N-BAR domain [39]. Alternatively, in the light of recent findings [40, 41], the loss of tubulation activity may only be due to a reduced membrane affinity of the mutant which leads to its reduced surface density and thereby results in impaired scaffolding activity. Endophilins have a third sub-module that is involved in membrane bending: an appendage of 30 amino acids (Q59-Q88), which is specific for the endophilin protein subfamily including nadrin, was shown to contribute to the induction of curvature [42]. On the sequence level, this appendage is inserted in helix 1 of the BAR domain and was found to protrude on the membrane-binding side from the center of the dimer [42]. Interestingly, an endophilin A1 BAR domain mutant where the entire appendage was replaced by a short helical stretch derived from the arfaptin2 sequence revealed structural integrity of the N-BAR dimer but showed reduced tubulation activity compared to the wild type [39]. Moreover, the mutant endophilin BAR domain-induced tubules with larger diameters, indicating that the appendage contributes to drive membrane curvature by inserting into the membrane like a wedge. Again, it has to be pointed at the importance of the surface density on the functionality of BAR domain proteins [43]. Therefore, data regarding the scaffolding activity of BAR domain proteins obtained by mutational studies can only be evaluated as solid when differences are seen at equal surface densities of mutant and wild-type proteins.

Mizuno et al. studied endophilin-coated membrane tubules by cryo-electron microscopy and identified different types of tubules depending on the concentration of the N-BAR domain [44]. They found chains of bulbous structures, quasi-cylindrical tubules of around 20 nm width and small tubules of 7 nm width which represent tubular micelles [44]. Reconstruction of the protein coat of quasi-cylindrical tubules showed that endophilin dimers pack in combined tip-to-tip and lateral inter-dimer manner. The latter are probably mediated by the amphipathic H_0_ helix. Mim et al. could show that the stability of the membrane-bound endophilin lattice is, indeed, largely conferred by dynamic interactions between neighboring H_0_ helices [45]. These H_0_:H_0_ interactions represent a fundamental difference in the lattice organization between N-and F-BAR domain proteins, as described below.

Two recent biophysical studies further contribute to our understanding of the membrane scaffolding mechanism by endophilin. Chen et al. [40] and Simunovic et al. [41] showed that the H_0_ helix was important for membrane recruitment of endophilin but—in contrast to Mim et al. [45]—did not find a significant contribution of the H_0_ helix to the membrane-curvature generation. Simunovic et al. further showed that the strongly curved endophilin initially assembled at the saddle-like base of a membrane nanotube and scaffold formation progressively emanated there from along the axis of the tubule [41]. In contrast, the initial assembly of centaurin, a protein with a shallow curvature of its BAR domain, was evenly distributed along the whole tubule [41]. Upon scaffold formation, the centaurin scaffolded tubule was four times wider than the endophilin scaffolded tubule which corresponds to the difference in the intrinsic curvature of their BAR domains. This study also indicated that scaffold formation does not require full protein packing but can occur already at lower surface densities of the BAR protein. In view of membrane processes like endocytosis, a less dense scaffold would leave sufficient membrane area for additional crucial membrane protein interactions.

#### F-BAR-domain: a coat for the membrane

The canonical view of BAR domains assumes that the curvature of the membrane-binding side is the main determinant of the diameter of induced membrane tubules. N-BAR domains with their highly curved concave shape, in general, form membrane tubules with smaller sized diameters than F-BAR domains [2, 39, 46] (Fig. 1a). However, tubules induced by F-BAR domain proteins are more variable in diameter. FCHo proteins induce highly curved tubules of about 20 nm or lower curved tubules of about 70 nm depending on their concentration in the liposome assay [47], whereas Cip4 induced tubules range from 60 to 80 nm [46].

Higher order oligomerisation of BAR-domain dimers and the formation of a helical lattice at the membrane has been investigated in two studies involving the F-BAR domains of FBP17 and Cip4 [26, 46]. Shimada et al. found filament like structures in the crystals where the F-BAR dimers associated via tip-to-tip interactions and could show by phase-contrast cryo-transmission tomography the striated structure of the F-BAR domain protein coat of a membrane tubule suggestive of stacked spirals of a protein filament wrapping around the tubule [26]. The tip-to-tip interaction involves residues T165 and K166 at the very tip of dimer, located in a turn between helices α3 and α4, and respective mutants result in impaired tubulation activity [26].

Apart from the tip-to-tip interactions, Frost et al. established that lateral interactions between neighboring FBP17 dimers are also essential for the assembly of helical lattices at membrane tubules [46]. Both hydrophobic (F276) and charged (K66) amino acids are involved in this inter-dimer lateral interaction. Using cryo-EM reconstructions of the protein coat of tubules with different diameters, they found a differential rotation of the F-BAR-domain dimers relative to the tubule`s cylindrical axis. This indicates that a differential assembly of F-BAR domain in a helical lattice rather than changes in the intrinsic curvature of F-BAR domains accounts for the various diameters of the membrane tubules induced by F-BAR domain proteins. Lateral inter-dimer interactions are the main contributors to the helical lattice formation in narrow tubules, whereas compromised lateral interactions lead to the formation of larger tubule diameters. Interestingly, Frost at al. also found that an F-BAR domain protein lattice can also form on flat membrane regions indicating that in this case another surface than the concave side of the BAR domain is involved in membrane binding [46]. The conserved residues K56, R104, K122, and K157 are likely to be involved in membrane interaction in this side-lying conformation and are associated with impaired tubulation activity when mutated. This finding may fuel some speculations on the process of membrane tubule formation by F-BAR domain proteins. An F-BAR protein coat may already assemble on a flat membrane region by forming a lattice of side-lying F-BAR dimers. A concerted rotation of the dimers that exposes the concave surface towards the membrane may then impose membrane bending and F-BAR protein lattice re-arrangement then results in final tubule formation.

As already discussed above, one determining factor of the membrane curvature is the variability in the assembly of the helical lattice that coats individual membrane tubules [46]. Another factor was elucidated for the F-BAR domains of the pacsin subfamily. Pacsin-1 and pacsin-2 induce membrane tubules of highly variable diameters, ranging from as low as 10 nm to more than 150 nm [48, 49]. In contrast, tubules formed by the subfamily member pacsin-3 were quite uniform with diameters around 100 nm. Bai et al. identified a proline (P121) within the so called wedge loop of pacsin-3 that conferred rigidity to this pacsin-specific structure and was responsible for its peculiar tubulation activity [49]. The wedge loop is a specific structural element of the pacsin subfamily that protrudes from the concave surface of the BAR dimer and is involved in membrane binding [50] (see blue spheres in Fig. 4). When the corresponding residues of pacsin-1 and 2 were mutated to prolines (Q124P, Q123P), the resulting mutant BAR domains likewise induced only low curvature tubules [49] suggesting that the wedge loop is involved in lateral inter-dimer interactions and filament formation. The flexibility of this loop in pacsin-1 and 2 may allow a high variability in filament assembly and thereby may account for the differently sized tubules generated by these proteins in biological processes like trans-Golgi network vesicle formation [14, 51] and caveola fission [12, 13]. Interestingly, over-expression of the pacsin-1 F-BAR domain does not only induce intracellular tubules but also microspike formation [52]. The F-BAR domains were localized to the neck of the microspikes, indicating that the concave membrane-binding surface of the F-BAR domain can stabilize the positive membrane curvature at the neck of the protrusion. This, however, implicates a longitudinal rather than a perpendicular orientation of the dimers with respect to the axis of the protrusion and predominantly lateral inter-dimer interactions rather than tip-to-tip filament formation [52].

Careful characterization of the tubule-forming process by F-BAR domain proteins in a liposome assay led to the identification of different classes of tubulating activity: FBP17 and Cip4 develop many protrusions simultaneously over the surface of the liposome, whereas PSTPIP1 and pacsin-2 induce only few but fast growing and much longer tubules which originate from a restricted part of the liposome [53]. Moreover, the bending rigidity of the FBP17/CIP4 tubules is higher than that of the PSTPIP1/pacsin-2 tubules [53]. This classification of tubulation activity observed in the liposome assay correlates with the phylogenetic proximity of these proteins and is suggestive for an evolutionary diversification of F-BAR proteins and thereby for an expansion of the cellular toolbox for membrane manipulation.

#### I-BAR-domains: an inverse-BAR mechanism

Experiments performed by Suetsugu et al. indicated that the I-BAR domain of IRSp53 induced membrane deformations in artificial liposomes that were significantly different from the thin protrusions induced by N- and F-BAR domains [5]. The formation of clusters of small buds at the surface of liposomes could be interpreted as a compensatory outward buckling of excess lipid bilayer caused by the I-BAR domain-induced inward membrane deformation of the spherical liposome. This interpretation was corroborated by the findings that the convex side of the I-BAR domain, rather than the concave side of the N- and F-BAR domains, conferred lipid-binding and that over-expression of the IRSp53 I-BAR domain-induced cell protrusions rather than intracellular tubules as found in F- and N-BAR domains over-expressing cells [5]. Using PIP2-enriched liposomes, Mattila et al. found that the I-BAR domain of IRSp53 induced tubular structures [8]. EM tomography of intact vesicular structures revealed that the tubules typically invaginated toward the interior of the vesicle indicating an inverse mechanism of membrane deformation [8]. Saarikangas et al. could show, by cryo-EM, that the tubules induced by the I-BAR domains of IRSp53 and MIM contained perpendicularly oriented striations at the inner leaflet which is, in fact, a strong support for a oligomeric assembly similar to F- and N-BAR domains but on the inside rather than the outside of membrane tubules [7]. I-BAR domains of MIM and ABBA induced tubules with significantly larger diameters than the I-BAR domains of IRSp53 or IRTKS, roughly 60 versus 40 nm. The phylogenetic distance between MIM and ABBA on one hand and IRSp53 and IRTKS on the other hand is much closer than that between respective members of these pairs of proteins. Co-expression of closely related I-BAR domains led to the formation of membrane tubules with similar diameter and co-segregation of these proteins, whereas a clear segregation into distinct filopodia or filopodial compartments was observed upon co-expression of distantly related I-BAR domain proteins [7]. Interestingly, an N-terminal amphipathic helix was found to render membrane binding und tubulating specificity to the MIM and ABBA I-BAR domains. The MIM mutants lacking this N-terminal helix show a salt sensitive membrane association and induce membrane tubules with significantly smaller diameters—both characteristics resemble the properties of the IRSp53 and IRTKS I-BAR domains [7]. Prévost et al. reported a phase separation process of IRSp53-loaded nanotubes at low protein densities resulting in the co-existence of protein-dense regions with low diameter and protein-bare regions with wide diameter [54]. This phase separation property of IRSp53 in the liposome assay is suggested to correlate with the tendency of this protein to form clusters in vivo. Interestingly, IRSp53 cluster formation at the plasma membrane was found to immediately precede filopodia growth indicating an important function for IRSp53 protein in the initiation of this membrane structure [55].

#### Tubulation versus vesiculation

An interesting antagonism between amphipathic helices and BAR domain scaffolds (and other scaffolds like clathrin coats) likely regulates membrane fission events, which are the final stage in intracellular vesicle formation processes [56]. The finding that ENTH domain-containing protein epsin and N-BAR domain proteins amphiphysin and endophilin not only generate membrane curvature, as the first step, but also promote membrane scission, as the last step in vesicle formation, suggested that amphipathic helices were involved in both processes. The shallow membrane insertion of an amphipathic helix expands the respective leaflet of the bilayer and induces a local positive membrane curvature [57]. A saddle shaped membrane neck that connects a nascent vesicle with the mother membrane has both negative and positive curvatures: amphipathic helix insertion destabilizes this membrane region and promotes the scission process, whereas a BAR domain protein coat wrapped around the tubular membrane neck stabilizes the region and counteracts scission. Using in vitro liposome assays as well as cellular over-expression and quantifying the ratio between generated tubules and vesicles, Boucrot et al. could show a positive correlation between the number of amphipathic helices and membrane fission activity in BAR domain proteins endophilin, amphiphysin, and GRAF, with four, two, or no amphipathic helices/helix per dimer, respectively [56]. Recombinant BAR domain proteins with additional amphipathic helices showed increased vesiculation activity, whereas recombinant endophilin with the amphipathic helices replaced by hydrophilic helices lost the vesiculation activity and mainly generated tubules. This study sheds light on mechanisms that are likely involved in the regulation of intracellular vesiculation processes and for example explains the necessity of a sequential and localized recruitment of various BAR domain proteins during the different stages of clathrin-dependent endocytosis [56].

An EPR study by Jao et al. indicated that the overall structure of the endophilin N-BAR domain is retained upon membrane interaction and that its concave surface does not deeply penetrate into the acyl chain interior of the membrane [42]. They further showed that the endophilin-specific appendage becomes an amphipathic helix upon membrane interaction. The respective helices of the dimer are antiparallel (and parallel to the membrane surface) and are located in the center of and largely perpendicular to the long axis of the dimer. This structure was, therefore, referred to as central insert region. Measuring the immersion depth of the membrane-interacting sub-domains into the lipid bilayer, Ambroso et al. found significant differences whether endophilin was associated with tubules or small vesicles [58]. On tubules, the H_0_ helix and the central insert region are deeply inserted into the acyl chain region of the lipid bilayer and the BAR domain is in contact with the lipid headgroups. On the other hand on small vesicles, only a shallow immersion of the H_0_ helix and the central insert region and no contact of the BAR domain with the membrane was found. This study indicates a different mechanism of curvature generation for vesiculation and tubulation. Wedging in the headgroup region by the H_0_ helix and the central insert region likely generate a splitting force between neighboring lipids thereby favoring vesiculation, whereas deep immersion allows the BAR domain to form a scaffold for tubules by contacts to lipids as well as high-order inter-dimer oligomerisation. Interestingly, the shift between tubulating and vesiculating activity of endophilin A1 is likely regulated by phosphorylation in vivo (see "A phosphosite in the endophilin-specific central insert region").

#### Curvature-sensing

Do the topology and the curvature of a membrane influence the recruitment of BAR domain proteins? BAR domain proteins were found to efficiently bind to small liposomes [2, 59, 60] and highly curved nanotubes [61], indicating that the intrinsic curvature of the BAR domain preferentially associates with membranes of higher positive curvature. Using the method of quantitative fluorescence microscopy to study curvature-selective binding proteins on liposomes of various diameters [62], Bhatia et al. found a strong curvature-sensing activity of the N-BAR protein endophilin A1 [63]. Interestingly, curvature-sensing of endophilin A1 was also effective at a concentration of 4 nM, where monomers are the dominant species. Moreover, comparison of membrane curvature-sensing of members of the N-, F-, and I-BAR domain subfamilies with their highly divergent shapes of the BAR-domain dimers, revealed similar curvature-sensing properties. These findings indicate that—while being essential for the membrane tubulating activity—the BAR domain dimer does not considerably contribute to curvature-sensing. Rather, the N-terminal amphiphilic helix of N-BAR domain proteins was identified as major determinant for curvature-sensing, probably by binding to membrane areas with lipid packing defects, a phenomenon that strongly increases with membrane curvature [62]. Similarly, as already mentioned, Chen et al. investigated the contributions of the amphiphilic helix of endophilin to curvature-sensing and scaffolding and found that it was important for membrane recruitment but dispensable for the scaffolding activity [40]. Interestingly, association of an arfaptin dimer and two Arl1 molecules results in the localization of this complex to highly curved membrane regions [64] (Fig. 1c). It is likely that the amphipathic helices of the Arl1 GTPases and the arfaptin dimer synergize to enhance the curvature-sensing property of the complex and thus drive its correct subcellular localization. Other in vitro studies revealed a curvature-selective affinity of BAR domain proteins for narrow membrane tubules [61, 65]. Using a giant unilamellar vesicle (GUV) with a highly curved membrane nanotubes pulled from them by optical tweezers, Sorre et al. found two regimes of membrane interaction of the N-BAR protein amphiphysin in dependence on the protein concentration [65]. At low protein densities, amphiphysin sensed the highly curved membrane region and accumulated at the nanotube, whereas at high protein densities, amphiphysin bound all over the GUV and induced tubule formation. Interestingly, centaurin, a BAR protein without any aliphatic helix, also sensed and accumulated at the highly curved membrane of the nanotube [41]. Likewise, IRSp53, an I-BAR protein lacking amphipathic helices, senses negative membrane curvature as shown by its sorting to the highly curved interior of nanotubes [54]. These data indicate that the BAR domain itself has a curvature-sensing property at least for tubular membranes. Extrapolating these findings to the cellular context, one can assume that curvature-sensing and lipid-binding specificity both contribute to the subcellular membrane targeting of a BAR protein and exceeding the surface density of the protein beyond a certain threshold and then initiates its scaffolding activity.

Indeed, a cell biological study is in line with the results of these biophysical investigations. Galic et al. used cell culture dishes spiked with cone-shaped nanostructures to mechanically induce an inward membrane curvature in adherent cells [66]. The N-BAR domain protein nadrin or its N-BAR domain alone was shown to be dynamically recruited to sites of nanocone-induced membrane invaginations. Similarly, inward membrane deformations induced by contracting actin cables specifically attracted N-BAR domain proteins nadrin and amphiphysin [65]. These findings again indicate that inward membrane deformations trigger the accumulation of N-BAR domain proteins via their curvature-sensing properties, resulting in the stabilization of these locally curved membrane regions.

#### The impact of membrane tension

Protein-driven membrane sculpting—be it by BAR-domain proteins or other membrane shaping proteins—has to be considered as only one factor in cellular membrane modeling processes. Lipid-modifying enzymes, which affect the local membrane lipid composition (of mostly only the inner membrane leaflet) and pushing or pulling forces of the local cytoskeleton, are other important players in this process. Indeed, apart from curvature-coupling proteins, membrane shape transitions are also induced by changed lipid composition or by lowering membrane tension [67]. Moreover, a recent simulation study indicated that membrane tension impacts the dynamics and geometry of BAR domain protein assembly [68]. Association of N-BAR proteins to a tensionless membrane strongly favors a linear protein aggregation with end-to-end inter-dimer couplings. Kinked end-to-end associations and side-to-side associations become more likely with increasing membrane tension. This together with increased protein density permits branching of protein aggregates and the formation of meshes. In an elegant liposome assay, Chen et al. investigated membrane-curvature transition in dependence of membrane tension and the density of BAR-domain proteins [69]. By assessing the maximal tension at which tubulation is possible, they find that I-BAR domains are stronger membrane-curvature generators than, e.g., the N-BAR-domain of endophilin. Further studies will be needed to detect additional features of BAR domains that govern the specific membrane shaping properties of the diverse members of this protein superfamily. Membrane shape transitions are likely co-regulated by the local density of a given BAR domain protein and the global membrane tension of the lipid bilayer. For further reading on that topic, we want to refer to the excellent review by Simunovic et al. where the impact of physical determinants in membrane modeling processes by BAR domain proteins is discussed [43].

## Functional diversification of BAR domains by other intramolecular domains

Most BAR-domain proteins contain several other functional domains. To date, little is known about the functional cooperativity and/or the regulatory influence between these domains in respective proteins. In this section, we will discuss structural studies that revealed close interactions between the BAR domains and their own intramolecular domains, conferring additional functional and/or regulatory mechanisms.

### The BAR-PH domain

BAR-domain protein subfamilies APPL, ASAP, centaurin β, and oligophrenin bear a PH domain C-terminally to their BAR domains, thereby suggesting a possible cooperativity between these two lipid-binding domains in membrane recognition (Fig. 2). Li et al. [70] and Zhu et al. [71] reported the crystal structures of the full length APPL1 and its BAR domain alone, respectively (Fig. 1b). The structure of the APPL1 BAR-domain dimer revealed some unique features as compared to other BAR domains. The BAR domain contains four instead of three helices, thereby generating a more extended interface between two APPL1 BAR subunits (Fig. 1b). In addition to the classical six helix bundle composed of two sets of the first three helices each, the fourth helix interacts with the first three helices of the other subunit. The two PH domains are in close contact with the BAR domain of the symmetry mate and located at distal ends of the dimer. The intramolecular association between the BAR and the PH domain was also corroborated by yeast two-hybrid interaction studies [70]. The N-terminal region does not form an amphipathic helix as in other N-BAR domain proteins but rather packs into a groove at the convex side of the dimer and forms a critical interaction site for the tight association of the PH domain. PH domains are known to fulfill major functions, namely lipid-binding and binding of small GTPases [72]. Interestingly, polar residues that have previously been identified to be involved in the phosphoinositide-binding are not conserved in the APPL1 PH domain. While the lipid-binding site of the APPL1 is still not unequivocally identified, Chial et al. showed phosphoinositide-binding of the full length APPL1 and APPL2 proteins as well as the PH domains alone [73].

King et al. determined the structure of BAR-PH domain of APPL2 and derived a model of the full length protein in solution using small angle X-ray scattering (SAXS) [74]. Similar to APPL1, they found several interaction regions between the PH domains and the BAR domain dimer. Interestingly, structural analysis suggested that the PH domain may rotate with respect to the BAR domain. Still awaiting further investigation, this finding may be of relevance for lipid-binding and the association to Rab GTPases. As for APPL1, the PH domain of APPL2 lacks the high affinity binding motif for phosphoinositides, but non-canonical binding sites exist [75] that likely confer membrane lipid association [74].

### The PX-BAR domain

The sorting nexins (SNX) form a subfamily of N-BAR domain proteins that are known to interact with endosomal components and play a role in endocytosis. The BAR domain is located at the C-terminal end of the protein and is preceded by a PX domain that is known to specifically bind to phosphoinositides (Fig. 2). SNX9, SNX18, and SNX30 are the only sorting nexins with an additional SH3 domain at their N-terminus. Pylypenko et al. solved the structure of the PX-BAR protein of SNX9 (residues 204–595) [60], which presents a crescent-shaped BAR domain dimer with the PX domains and small sub-domains termed “yoke domain” or “Y domain” symmetrically flanking the tips of the BAR domain dimer (Fig. 1b). In contrast to the BAR-PH domain, where a direct interaction of both domains occurs, the Y domain interconnects the PX domains and the BAR domain dimer. The PX domain is sandwiched between two Y sub-domains [214–250 (Y_N_)) and (375–390 (Y_C_)]. The evolutionary conserved residues 201–213 that precede the Y_N_ domain form an amphipathic helix upon membrane binding and are essential for the tubulating activity of the PX-BAR superdomain. Thereby, this arrangement is functionally similar to the amphipathic helix in N-BAR domain proteins. Liposome-binding studies revealed that the binding of the PX-BAR protein was independent of the liposome size in the presence of phosphoinositides, suggesting a strong tubulating capacity. Conversely, when phosphoinositide-free liposomes were used, the PX-BAR domain only bound to liposomes smaller than 100 nm, indicating a clear curvature-sensing effect [60]. Interestingly, SAXS analyses of SNX9 showed that the PX-BAR dimer adopts a more curved conformation in solution than in the crystal [76]. Moreover, the PX domain revealed a considerable mobility relative to the BAR domain, while the N-terminal SH3 domain was suggested to influence the conformation and probably also the mode of oligomerisation of SNX9 [75]. These data indicate a complex interplay of the domains that govern the functional mode of SNX9. The current model of the SNX9 membrane-binding mechanism likely includes the following steps [60]: (1) sequential binding to PIPs via the two phosphoinositide-binding pockets of the PX domain leading to (2) the tightening of the interaction between PX and BAR domains; (3) membrane insertion of N_0_ amphipathic helix that induces membrane curvature; and (4) lateral oligomerization of PX-BAR domains to promote tubule formation. Further studies will be necessary to elucidate the molecular details of the role of SNX9 in endocytosis where SNX9 proteins are likely recruited to clathrin-coated membrane buds via PIPs and then exert their tubulating activity to generate the narrow neck prior to the final dynamin-dependent vesicle scission event.

### The PDZ-BAR domain

The scaffold protein PICK1, which consists of a PDZ domain, a central BAR domain, and a C-terminal ACT domain, is predominantly expressed in neuronal cells and has been recognized in trafficking of important neuronal proteins. PDZ domains are interaction modules implicated in binding short motifs at the C-termini of target proteins [77]. Two concurrent studies reported on molecular models of PICK1 using the SAXS analysis [78, 79]. Both investigator groups faced the same problem—sample aggregation, which can impair or bias structural investigations and conclusions.

Madasu et al., who circumvented the aggregation by recombinantly fusing the human PICK1 to the C-terminus of the maltose-binding protein (MBP), used SAXS to analyze the full length PICK1 molecule [78]. Modeling of the MBP-PICK1 dimer into the elongated banana-shaped SAXS envelope positioned the BAR domain dimer in the middle and the MBP at the ends of the envelope (Fig. 1b). The PDZ domains were located at the distal ends of the BAR domain dimer, indicating a tight association between the PDZ and the BAR domain (Fig. 1b). This region was also predicted to contain an α-helix (helix 2) with positively charged residues which was modeled to become part of the membrane-binding surface on the concave face of the BAR domain. In contrast to the well-constraint position of the PDZ domain on the PICK1 dimer tails, the orientation of its ligand-binding site could not be derived from the model [77]. Remarkably, mutational analyses suggested that the positively charged residues in helix 2 contribute to the membrane association of PICK1 [80]. It is likely that membrane and receptor binding occur at the same side of the dimer that allows for a “coincidence detection” mechanism as suggested [80]. Thus, these studies indicate a rigid assembly of a PDZ and BAR domains that presumably targets PICK1 to receptor-rich membrane sites.

Karlsen et al., on the other hand, generated a mutant (PICK1^LKV^) in which the last three amino acids of PICK1 (^413^CDS^415^) were replaced by the specific sequence LKV that binds in the pocket of the PDZ domain, alleviating to some extent the aggregation issue [79]. They analyzed the SAXS data using the decomposition approach, assuming that the sample consisted of dimers and tetramers. The detailed analysis of the dimeric portion of the decomposed data suggested that the BAR and PDZ domains were well separated from each other and connected by a flexible linker, and that the structure can best be presented with a conformational ensemble (Fig. 1b). The authors further extended the analysis on tetramers and proposed an oligomerisation model involving an offset between the individual BAR domains [78].

The two structural studies on PICK1 differ both in the structure of the PICK1 dimer as well as in findings about the higher oligomerisation state. The compact structure by Madasu et al. [78] seems to be in better agreement with the circumstantial data which suggest that the PDZ domain is in direct contact with the BAR domain [81], that the linker between the PDZ and BAR domain has a strong helical propensity, and that the PDZ domain participates in membrane binding where it has an auto-inhibitory role, regulating BAR domain interactions with other proteins [82]. This can be envisaged if the PDZ and BAR domains lie adjacent to each other. Albeit both models are profoundly different, the compact model does not a priori exclude flexibility within the PICK1 molecule, awaiting alternative structural and biophysical approaches to address this question.

### Regulation by an acidic C-terminal tail

As outlined above, PICK1 is composed of a conserved PDZ and BAR domains, and a less conserved acidic C-terminal tail (ACT) of about 60 amino acids. Secondary structure analyses predict that ACT is mainly disordered and modeling of the SAXS data indicates that it protrudes from the PDZ-BAR surface of the second subunit of the PICK1 dimer [78]. The functional relevance of ACT for subcellular localization of PICK1 was shown by over-expression studies, where wild-type PICK1 was found throughout the cytoplasm, in contrast to a punctuate membrane association in constructs lacking ACT. Thus, the acidic residues in the ACT domain likely interact with the basic residues at the concave side of the BAR domain. ACT may thereby work as a flexible regulator that affects membrane association of PICK1. Receptor binding by the PDZ domain likely induces a conformational change that abolishes the auto-inhibitory effect of ACT and allows membrane association by the BAR domain [80, 83–85]. Thus, receptor-dependent localized membrane association of PICK1 is likely regulated by its acidic C-terminal tail.

### Auto-regulation by the SH3 domain

Endophilin is one of the BAR domain proteins that play a central role in endocytosis. It is composed of an N-BAR and a C-terminal SH3 domain, a domain typically binding to proline-rich peptides of respective binding partners (Fig. 2). SAXS analyses of the full length protein in solution revealed that the SH3 domains were located at the tips of the BAR domain dimer [76], similar to the PH domains in APPL1 [71]. Computer simulations predicted that the SH3 interacts with the N-terminal amphipathic helix H_0_, which is thought to be unstructured in solution and only becomes helical when the molecule is in contact with the membrane. The role of the interdomain interaction between the SH3 and the BAR domain was suggested to be the stabilization of the H_0_ helix in solution [86] by protecting its hydrophobic residues. Moreover, the electrostatic potential of the H_0_/SH3 complex is separated in positively and negatively charged regions with the positively charged residues of the amphiphilic helix located at the same side as the concave membrane-binding side of the BAR domain. The H_0_/SH3 complex formation was corroborated by kinetic studies of endophilin dimer dissociation where mutants lacking either the H_0_ helix or the SH3 domain revealed significantly faster dissociation kinetics than the full length protein [87]. The data further indicate that each SH3 domain cross reacts with the H_0_ helix of the juxtaposed subunit of the dimer. Thus, the interaction between the SH3 domain and the H_0_ helix implies a dual auto-regulation of the endophilin: the H_0_ helix and the SH3 domain are both inhibited from membrane binding and association with SH3 binding partners, respectively. In line with this model, Meinecke et al. showed that membrane association of endophilin and amphiphysin, another N-BAR and SH3 domain-containing protein, was dependent on the presence of SH3-binding partner dynamin, a GTPase essential for vesicle scission [88]. This auto-regulation of endophilin and amphiphysin thereby ensures a reciprocal recruitment of essential factors to endocytic membrane sites [87].

A similar auto-regulatory property was described for the F-BAR domain protein syndapin1/pacsin-1 [48]. The strong tubulating activity of the F-BAR domain in liposome assays was considerably impaired when full length pacsin-1 was used. In vesicle pelleting assays, the curvature preference was restricted to large vesicles in full length pacsin-1, whereas the F-BAR domain alone also bound to small vesicles. Moreover, expression of full length pacsin-1 in Cos7 cells resulted in predominantly cytoplasmic localization of the protein and no vesicle or tubule formation, whereas the isolated F-BAR domain alone produced several tubules from perinuclear membranes [89]. The crystal structure of the full length pacsin-1 clearly revealed the structural basis of this regulation [88]. The SH3 domain contacts the tips of the F-BAR domain at the concave—the membrane binding—side and the PxxP peptide-binding groove of the SH3 domain was buried in the contact area (Fig. 1b). The interaction is mainly conferred by salt bridges and hydrogen bonds and is likely weaker than the association between the SH3 domain and its binding partners, as it does not engage the groove in which SH3 ligands typically bind, suggesting that the interdomain complex is the preferred state of the pacsin-1 dimer in solution but is readily broken in the presence of appropriate binding partners. In conclusion, the interdomain association between the SH3 domain and the BAR domain seems a general auto-regulatory feature of respective N-BAR and F-BAR domain proteins.

## Protein interactions of BAR domains

### Arfaptin binds small GTPases

The Ras superfamily is a class of small GTPases that act as molecular switches in a wide variety of cellular functions. Interestingly, the BAR domain protein arfaptin was shown to interact with several members of this large protein family, namely with Rac1, thereby being involved in membrane ruffling at the plasma membrane, as well as with GTP-bound ADP ribosylation factor (Arf) proteins and the Arf-like protein Arl1, implicating a regulatory function at the Golgi complex [90–92]. Tarricone et al. reported the crystal structure of the N-BAR domain of arfaptin2 in complex with the GDP-bound form of Rac—in fact, this was the first structural analysis of a BAR domain [93] (Fig. 1c). In the complex, arfaptin dimer binds one Rac molecule, which is positioned centrally to the concave side of the crescent-shaped arfaptin dimer. Funnily enough, the nowadays well-established membrane-binding side of a BAR domain was at first analyzed for a specific alternative property—as a site for protein–protein interactions! Competition experiments indicated that the binding of GDP Rac and GTP Arf to arfaptin is mutually exclusive, thereby suggesting an overlap of the respective binding sites. Support for a competition in binding of Rac1 and Arf to arfaptin came from structural analysis of the complex of Arl1 and the BAR domain of arfaptin2 that revealed different small GTPase-binding sites at the BAR domain dimer of arfaptin [94] (Fig. 1c). The Arl1-arfaptin complex is similar to the structure of the PX-BAR domain with one Arl1 molecule being laterally associated at each side of the arfaptin BAR domain dimer. A quantitative binding study confirmed the structural prediction that simultaneous binding of Rac1 and two Arl1 molecules by one arfaptin dimer was incompatible; however, a complex of arfaptin dimer with one molecule of Rac1 and one of Arl1 would be possible. These data further indicate that Arl1 association does not compete with the membrane-binding activity of the BAR domain; rather it is likely that the association with Arl1 enhances membrane binding because of the membrane anchoring property of the myristoylated amphipathic helices at the Arl1 N-terminus. Moreover, binding to Arl1 recruits arfaptin2 to the trans-Golgi network, where it colocalizes with vesicular and tubular structures [95]. Similarly, the association with Arf1 has been shown to enhance the recruitment of arfaptin to curved membranes in a liposome assay [64]. Thus, the lateral association of Arl and Arf GTPases at the BAR domain specifies the membrane localization of arfaptin.

### Rac binds to I-BAR domains

Studying the complex regulation of membrane ruffling Miki et al. identified IRSp53 as a novel Rac-binding protein [96]. Using pull-down assays, the smallest binding fragment of activated Rac comprised the N-terminal 229 amino acids and was hence termed Rac-binding domain (RCB)—this was the first name of the domain which is now most commonly termed I-BAR domain. Full length IRSp53 has a reduced Rac-binding ability due to an auto-inhibitory mechanism probably mediated by the SH3 domains; however, complex formation of IRSp53 and WAVE2 enhances the Rac-binding efficiency of IRSp53 [97]. Suetsugu et al. identified amino acids in the RCB/IMD/I-BAR domain that are crucial for Rac-binding and assessed the dissociation constant of the complex of RCB domain and GTPγS-loaded Rac to be about 3 µM—a value comparable to that of the complex between arfaptin and Rac GMPPNP [5]. The GDP-loaded Rac RCB complex had a much higher dissociation constant of about 20 µM. Liposome assays further revealed a partial competition between Rac- and membrane-binding of the RCB domain only when Rac was devoid of lipid modifications. However, Rac bearing a lipid modification binds to the membrane-associated RCB/IMD/I-BAR domain. The interaction between the I-BAR domain of IRSp53 and Rac1 is part of a complex molecular network that regulates the formation of membrane protrusions and the migration of macrophages [98].

Rac was also found to bind to MIM-B, another I-BAR domain protein with a regulatory role in cytoskeleton-based processes [99], again specifically interacting with its I-BAR domain. This domain was also shown to activate Rac, however, a direct GTP exchange factor (GEF) activity of this domain was ruled out. Further studies are needed to fully evaluate the regulatory role of the Rac MIM-B interaction.

### The BAR domain of APPL interacts with Rab GTPases

The APPL proteins contain a BAR-PH domain module and a C-terminal phosphotyrosine-binding domain (PTB) and are known Rab effectors functioning in nuclear signal transduction (Figs. 1b, 2). Miaczynska et al. found that the active, GTP-bound form of Rab5 binds and targets APPL1 to a specific type of endosomal vesicles and that GTP hydrolysis releases APPL1 there from and enables its nuclear translocation [100]. Two Rab5-binding sites were identified at opposite ends of the BAR-PH dimer and a dissociation constant of 0.9 µM—typical for interactions between Rab and effector proteins—was determined. Mutational analysis guided by the APPL1 BAR-PH structure revealed that the Rab5 binding site is mainly contained within the PH domain and the BAR domain also contributes to the association indicating a BAR-stabilized PH domain as essential for Rab5 binding [71]. Rab21, a member of the Rab5 subfamily, was also shown to bind to APPL1, however, with a slightly different binding profile suggesting a functional diversity of APPL1 in the interaction with different Rab proteins. Moreover, the surface of Rab5 for binding to APPL1 differs from that for binding to other Rab5 effectors. Thus, the specific association between the Rab proteins and BAR-PH domain of APPL1 represents a novel-binding mode for this type of GTPases [70]. APPL2, a close relative of the APPL1 protein, also binds Rab5, but does not interact with Rab21, rather it interacts with Rab22a, Rab24, and Rab31 [74]. This indicates that a co-evolution of APPL proteins and their specific Rab-interaction partners has taken place.

### A GTP exchange factor binds endophilins N-BAR domain

**Fig. 3:**
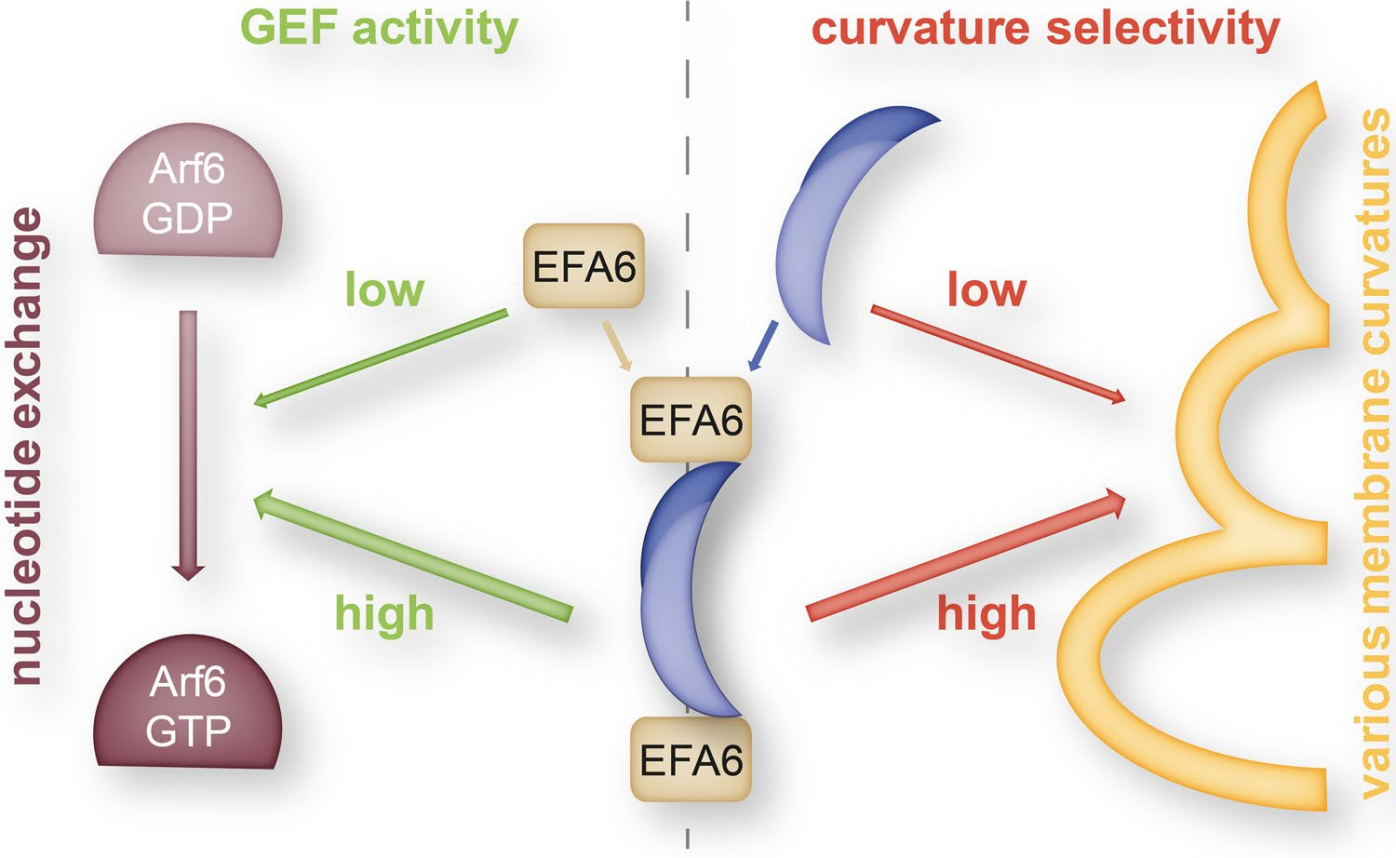
Complex between endophilin and EFA6 as regulator in clathrin-mediated endocytosis. The N-BAR domain of the endophilin dimer (*dark* and *light blue* overlapping moons representing endophilin monomers) interacts with the Arf6-specific exchange factor (EFA6). The complex exhibits both increased guanine-nucleotide exchange factor (GEF) activity of the EFA6 constituents and increased selectivity of the endophilin dimer for highly curved membrane shapes (*yellow*) and thereby plays a crucial role in orchestrating the sequential steps (Arf6 activation and selective membrane tubulation) in clathrin-mediated endocytosis [101]

Another interesting functional link between the superfamilies of BAR domain proteins and small Ras-like GTPases was found by Boulakirba et al. reporting the direct association of EFA6A (the Arf6-specific exchange factor) and endophilin [101]. Using the catalytic Sec7 domain of EFA6 as bait in a two-hybrid screen, several clones of endophilin B1 were isolated; however, also endophilin isoforms A1 and A2 were shown to interact. The interacting region was pinned down to the first 125 amino acids of the N-BAR domain of endophilin. Interestingly, the interaction with endophilin greatly enhanced the GEF activity of EFA6A as seen in an Arf6 activation assay. In turn, the interaction with EFA6A also modulated the membrane binding and tubulating activity of endophilin. The presence of EFA6A impaired the binding of endophilin to large liposomes (mimicking flat membranes) but not to small liposomes of 50 nm diameter (mimicking endocytic vesicles). Similarly, EFA6A interfered with the tubulating activity of endophilin at large but not at small liposomes. Thus, it is likely that EFA6A binds to the concave side of the endophilin dimer and that this interaction is abrogated in the presence of curved membranes suggesting membrane-curvature-dependent protein interaction. It was further shown that co-expression of endophilin together with EFA6A clearly changed its localization from the cytoplasm to EFA6A—positive membrane ruffles. Together, these data suggest a sequence of events for endophilin-dependent and clathrin-independent [11, 102, 103] endocytosis: EFA6a recruits endophilin to flat membranes where they cooperate to activate Arf6 which, in turn, initiates membrane budding for clathrin-coated vesicle formation (Fig. 3). The curved membrane then dissociates the endophilin-EFA6A complex and endophilin can promote endocytic vesicle fission.

### Binding of BAR domain dimers to actin

A tight spatial and temporal coordination of actin polymerization and plasma membrane remodeling is a characteristic feature of many cellular processes, including endocytosis, exocytosis, cell motility, and intracellular trafficking. In these processes, BAR domain-containing proteins emerged as key regulators linking signaling pathways to actin cytoskeleton and membrane dynamics. To regulate actin dynamics, several members of BAR domain family directly bind to actin, or to actin-associated and regulating proteins via distinct domains. In this respect, BAR domain proteins were shown to bind to both monomeric (G-actin) as well as filamentous (F-actin) actin. While binding to G-actin is mediated through specialized G-actin-binding domains (e.g., WH2), F-actin binding was shown to be mediated directly through their BAR domains. Until now, binding to F-actin was demonstrated for six BAR domain proteins, namely Gas7, PICK1, MAYP/PSTPIP2, pacsin-2, MIM, and IRSp53 [6, 8, 50, 104–106]. Thus, all types of BAR domains (N-, F-, and I-BAR) were shown to be involved in the interaction with actin. Binding of BAR domains to actin is mediated by positively charged residues (Table 1), which map (mostly) to the concave side of the molecule, and overlap with the binding site for membrane lipids. In vitro, these domains bind F-actin with affinities ranging from 0.3 to 17 µM (Table 1), which are similar to other actin-binding proteins. In all cases, this interaction is salt dependent, thereby underlining the electrostatic nature of this interaction [6, 8, 50, 104–106]. In addition, binding of these domains to actin was shown to have an effect on actin dynamics and stability (Table 1). While binding of BAR domains to actin in vitro is quite established and characterized (e.g., binding of pacsin-2 to actin [50], see section 5.6.1), evidence for binding of these domains to the F-actin in vivo is controversial. Several studies showed that these BAR domains do not colocalize with F-actin in cells, and the interactions are suggested to depend on non-specific electrostatic contacts [8, 78].

**Table 1:** Characteristics of F-actin-associating BAR domain proteins

Protein	Domain type	Affinity to F-actin (*K*_d_)	Interaction site	Function (in vitro)^a^	Function (in vivo)^a^
PICK1	N-BAR	0.3 µM [105]^b^ 2.0–3.0 μM [78]^c^	BAR domain K251, K252 [105]	1. Inhibits Arp2/3-mediated actin nucleation [105]^d^ 2. Binds to F-actin, but it neither binds nor inhibits Arp2/3 complex [78]	1. Contribution to specific form of vesicle trafficking, and the development of neuronal architecture [105] 2. No colocalization and binding with actin in vivo [78]
Gas7	F-BAR	0.3 µM [104]^e^	BAR domain [104]^f^	Promotes actin assembly and crosslinks actin filaments [104]	Reorganization of microfilaments and promoting of membrane outgrowth [104]
PSTPIP2	F-BAR	N.D	BAR domain [106]^f, g^	Induces actin bundling, reduces the rate of actin polymerization, and increases its stability [106]	Effects on actin bundling and filopodia formation, and directional migration [106]
Pacsin-2	F-BAR	2.0 μM [50]	BAR domain mainly two clusters of lysine residues [50]^h^	Increases the stability of F-actin [50]	N.D
MIM	I-BAR	17 µM [107]	BAR domain basic residues at the distal ends of the dimer [8] ^i^	1. Bundling of actin filaments [99, 110, 111] 2. Weak or no bundling activity [8, 107]	1. Filopodia/microspike formation [99, 111] 2. Does not contribute to filopodia formation [8]
IRSp53	I-BAR	5 µM [6]	BAR domain basic residues at the distal ends of the dimer [6]^i^	Bundling of actin filaments [6, 111]	Filopodia formation [6]

*N.D*. not determined

^a^Function related to F-actin binding

^b^Kd determined for the full length protein; isolated BAR domain bound to actin more efficiently than full length PICK1

^c^Kd determined for the full length protein

^d^Through interactions of the BAR domain with F-actin and the ACT with Arp2/3

^e^Determined indirectly by comparison with α-catenin

^f^Detailed mapping was not done

^g^Experiments were done with the full length protein, which does not have any other domain than BAR

^h^Indentified by cross-linking; however, some other residues might be involved as well

^i^Other parts of the I-BAR are most likely also involved

Indeed, PICK1 was initially found to bind to F-actin and the Arp2/3 complex and thus to inhibit Arp2/3-mediated actin assembly activated by the N-WASP VCA domain, which is important for morphology of neurons and endocytosis stimulated by AMPA receptor [105]. In a recent study, however, PICK1 was found neither to bind nor to inhibit the Arp2/3 complex. In addition, although PICK1 was confirmed to interact with F-actin in vitro, its co-localization with F-actin in cells was not observed [78].

Similarly, earlier studies demonstrated that I-BAR domain of MIM binds and bundles actin filaments, interacts with the small GTPase Rac, and thus, it is important for MIM filopodia-forming activity [99]. Later on, however, it was shown that the I-BAR domain of MIM displays only very weak [8] or no F-actin bundling [107] activity at physiological conditions and thus likely does not contribute to filopodia formation in vivo. In agreement with this observation, analysis of filopodia from cells expressing GFP-tagged I-BAR domain of MIM revealed that the I-BAR domain does not localize to the F-actin bundles [8]. In comparison to other BAR domain proteins which interact with F-actin through their BAR domains, MIM possesses a C-terminal WH2 domain (Fig. 2), which binds actin monomers with high affinity [108, 109]. Thus, although the affinity of I-BAR domain of MIM towards F-actin is low, its presence at high local concentrations of actin, to which it is recruited via its WH2 domain (the MIM/ATP-G-actin complex can participate in actin filament assembly at the barbed end [108]) which can lead to its increased association with F-actin. This is supported by the fact that full length MIM binds F-actin stronger (0.15 µM [110]) than I-BAR domain alone (17 µM [107]). Hence, it remains to be elucidated how much the suggested “non-specific” interaction of I-BAR domain of MIM with F-actin [8] exerts an effect when being in close proximity to F-actin in vivo.

The situation might be more complex for IRSp53, which possesses an N-terminal I-BAR domain structurally, sequence wise and functionally related to the I-BAR domain of MIM as well as a C-terminally located WH2 domain (Fig. 2). However, besides these two domains, IRSp53 has a centrally located SH3 domain, through which it is linked to the Arp2/3-mediated actin filament assembly (Fig. 2). Similarly to MIM, the I-BAR domain of IRSp53 was shown to bind and bundle F-actin via basic residues at the extreme ends of the I-BAR dimer (Table 1), and thus be involved in the filopodia formation in vivo [6, 111]. However, specificity and/or non-specificity of this interaction in vivo, as shown for MIM, still need further experimental confirmation.

The same is true for Gas7, the over-expression of which leads to changes in microfilament organization. Gas7 co-localizes with F-actin in membrane ruffles and was found to interact and cross-link actin filaments in vitro via the C-terminal BAR domain [104]. However, its binding to F-actin in vivo was not confirmed and yet its effects on cell morphology and its association with actin can be indirect, via its N-terminally located SH3 domain (Fig. 2). In support of this notion, neither SH3 nor BAR domain alone were found to be sufficient to induce the cell morphology changes observed after over-expression of full length Gas7 [104].

This, however, is not the case for PSTPIP2, which was found to associate with actin in macrophages [112]. In vitro, PSTPIP2 was shown to induce F-actin bundling, reduce the rate of actin polymerization, and increase its stability [106]. In vivo, PSTPIP2 has an effect on the organization of the actin cytoskeleton leading to affected macrophage morphology and motility, and co-localizes with F-actin at the bases of filamentous protrusions [106]. However, while other F-actin-associating BAR domain proteins possess, in addition to their BAR domain, other domains mediating their direct (WH2 domain) or indirect (SH3 domain) binding to actin, PSTPIP2 is composed solely of an F-BAR domain (Fig. 2). Hence, the effects of PSTPIP2 on actin in vivo and in vitro are mediated most likely by its BAR domain alone.

A general issue with the binding of BAR domains to F-actin can be seen in the fact that the interaction with F-actin and membrane lipids seems to be mutually exclusive. The affinity of the F-BAR domain of pacsin-2 is higher for membrane lipids than for F-actin, a finding that is likely also true for other F-actin-binding BAR domains [50]. Thus, in the presence of membranes, especially during the formation of vesicles or membrane protrusions, binding of BAR domains to F-actin would be reduced or not existing. On the other hand, many of the BAR domain proteins are specifically recruited to the actin cytoskeleton via specialized domains like WH2 or SH3 and the local vicinity may thus favor the association of the BAR domain with F-actin. It is conceivable that F-actin binding of BAR domain protein may be relevant at sites of active membrane remodeling, where iterative membrane association and dissociation of BAR domain proteins are known to take place (e.g., at endocytic sites). There, F-actin may sequester membrane-dissociated BAR-domain proteins until (regulated) re-association with the membrane is again required. However, high-resolution in vivo imaging studies will be necessary to address this question.

**Fig. 4:**
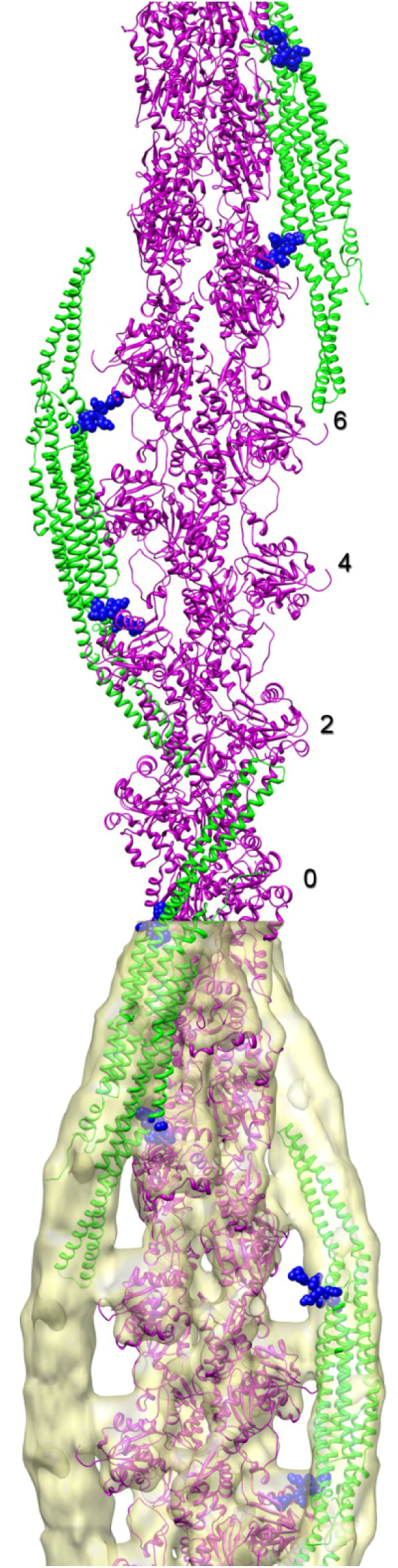
Model for pacsin-2 bound to F-actin, based on EM reconstruction of F-actin decorated by pacin-2 [50]. Actin subunits (*magenta ribbons*) are numbered along one strand. The two *green* pacsin-2 ribbons on the *right* bind to that strand. The *green* pacsin-2 ribbon on the *left* binds to the opposite actin strand. The *yellow* surface at the *bottom* is a three-dimensional reconstruction of the atomic model shown, after imposing the actin helical symmetry and filtering to 12 Å resolution. Residues of the wedge loop, pointing towards F-actin, are represented as *blue spheres*

In our recent study, we compared several BAR domains (F-BAR domains of pacsin-2, CIP4 and FCHO2 and N-BAR domain of enodphilin) with respect to their actin-binding properties in vitro [50]. Interestingly, F-actin binding was not a general property of the tested BAR domains but was rather specific for pacsin-2, probably due to the distinct pattern of positively charged patches within its BAR domain. This observation is suggestive of specificity that may be of relevance also in the context of cellular processes. In addition, helical reconstruction (cryo-EM) of the F-actin-pacsin-2 complex revealed that the F-BAR domain of pacsins binds along the long-pitch strands of the actin filament in a manner reminiscent of the tropomyosin F-actin interaction (Fig. 4). In a previous study, the co-localization of pacsin-2 with actin in CEHF cell was observed [113]; however, this interaction could be mediated indirectly through filamin A, which binds to F-BAR domain of pacsin-2 or through the SH3 domain of pacsin-2, which binds to N-WASP [113, 114], an important regulator of actin cytoskeleton organization [115]. Thus, the occurrence of a direct association of pacsin2 with F-actin in the cellular context still awaits to be shown.

### Pacsin interactions

#### Caveolin-1 binds to the F-BAR domain of pacsins

In 2011, two papers independently reported a role for the F-BAR domain protein pacsin-2 in caveolar biogenesis [12, 13]. Senju et al. found a direct interaction between caveolin-1 and pacsin-2 and mapped the interaction regions to the convex side of the F-BAR domain and the N-terminal cytoplasmic region (residues 61–100) of caveolin-1, respectively [13]. In contrast to pacsin-1, pacsin-3 also bound to caveolin-1; interestingly, pacsin-2 and -3 share a series of conserved residues that are exposed at the convex sides of their F-BAR domains. Neither membrane binding nor membrane tubulation activities of the F-BAR domain were impaired by the association with the caveolin fragment in in vitro assays. Conversely, the full length pacsin-2 protein revealed a considerable increase in both activities in the presence of the caveolin peptide. This indicates that caveolin-binding interferes with the intramolecular/intradimer associations between the SH3 and the F-BAR domain and thereby decreases the auto-inhibition of full length pacsin-2 [13]. Moreover, these membrane activities were further increased by the additional presence of the dynamin-derived PxxP peptide which binds to the SH3 domain of pacsin-2. Thus, a model of caveolae biogenesis includes a role for the BAR-domain in membrane sculpting and recruitment of dynamin-2 by the SH3 domain of pacsin-2 for the scission process.

#### Polycystin-1 interacts with pacsin-2

Polycystin-1 (PC1) is a large integral membrane protein involved in tubule formation in the major organs of our body and is associated with polycystic kidney disease (PKD), the most common life-threatening genetic disease. Yao et al. reported the interaction of the intracellular C-terminal domain (ICD) (residues 4079–4302) of PC1 with of pacsin-2 [16]. The pacsin-2 truncation mutant comprising residues 171–278 specifically interacted with PC1. This construct contained the α3 helix which is conserved among the F-BAR domains of the pacsin protein family and 39 residues of the linker region. It is yet not clear whether α3 helix alone is responsible for the interaction or whether the part of the linker region is also involved. A coiled-coil domain within the ICD of PC1 was shown to confer the interaction with pacsin-2. Interestingly, an ICD construct containing a pathogenic PKD mutation within the coiled-coil region failed to bind to pacsin-2. Moreover, pacsin-2 co-localized with PC1 at the lamellipodia of a mouse kidney cell line and was found by immunoprecipitation studies from cell extracts to be in a complex together with PC1 and N-Wasp. The SH3 domain of pacsin-2 is known to bind to and activate N-Wasp which, in turn, regulates actin nucleation via the Arp2/3 complex [116, 117]. Thus, pacsin-2 is involved in the regulation of cell migration and tubulogenesis by linking PC1 to the cytoskeletal network dynamics [118].

#### Filamin A promotes membrane tubulation by pacsin-2

**Fig. 5:**
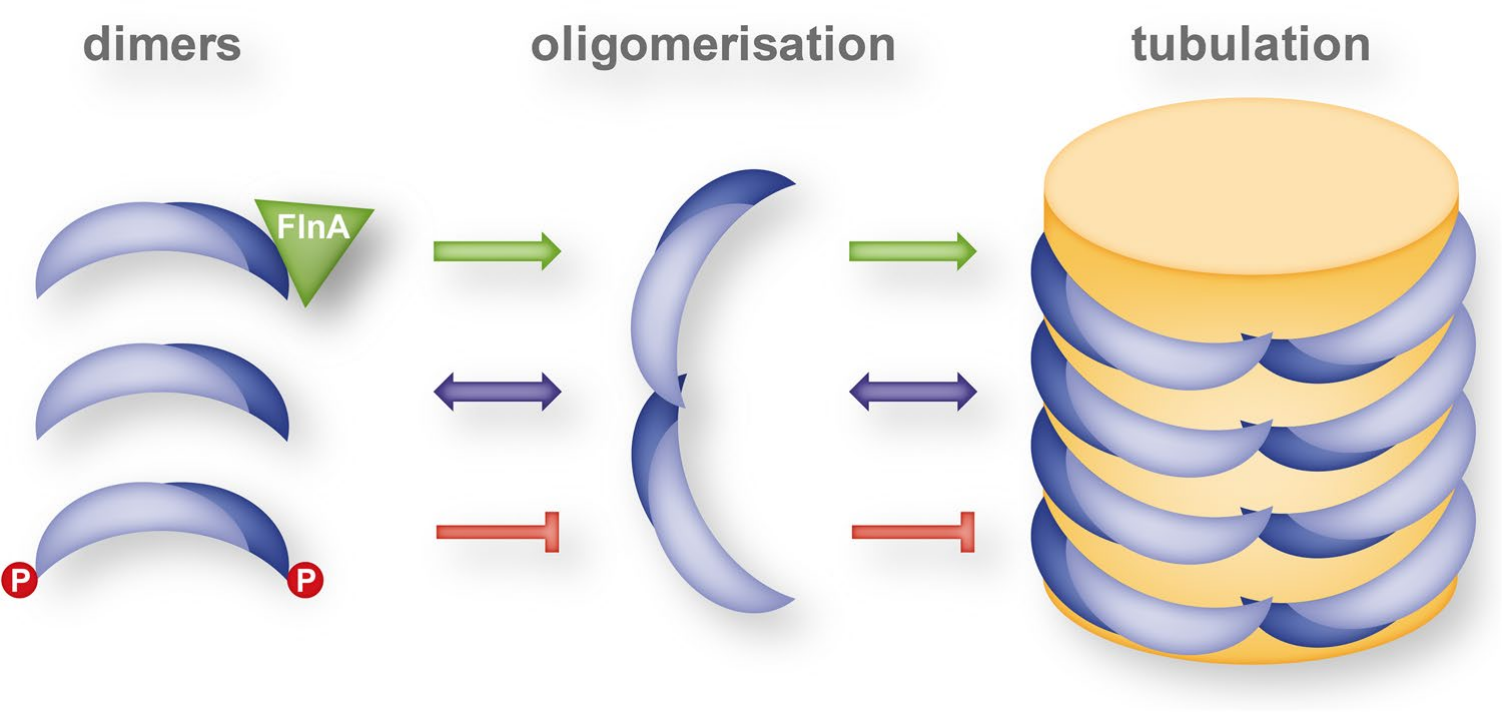
Regulation of pacsin’s membrane activity. Inter-dimer tip-to-tip oligomerization and membrane tubulation are reversible processes dependent on the pacsin dimer (*dark* and *light blue* moons) concentration. Phosphorylation at T181 (*red circles*) located at the tips of the dimers inhibits [137], whereas the presence of filamin A (FlnA) promotes [114] oligomerisation and pacsin-dependent membrane tubulation

The interaction between pacsin-2 and filamin A (FlnA), a cytoskeletal and scaffold protein, was first identified by Nikki et al. implicating a role for this complex in regulation of cytoskeletal processes at focal adhesions [113]. This complex was further found in podosomes and in cell adhesion during gastrulation in *Xenopus laevis* [119, 120]. Recently, an eminent role was revealed for membrane tubulation processes in megakaryocytes and platelets where pacsin-2 is very abundant [114]. FlnA is 280 kDa protein composed of an N-terminal actin-binding domain, 24 immunoglobulin-like domains, of which the C-terminal domain is responsible for dimerisation. Jurak Begonia et al. mapped the pacsin-2-binding site to FlnA domain 20, which is close to the binding sites for GPIbα and integrin β1 in domains 17 and 21, respectively [114]. A potential FlnA-binding motif was mapped to the 174–182 pacsin-2 region, as already earlier suggested [113]. This motif is located at the tips of the pacsin-2 dimer in a loop between helix α2 and helix α3 [113]. An atypical proline residue at position 180 within the FlnA-binding motif was shown to be necessary to confer binding to FlnA domain 20, as the P180A pacsin-2 mutant abolishes binding to FlnA. As the tips of the pacsin dimers are known to be involved in inter-dimer association during the membrane tubulation process, the impact of FlnA binding for the tubulation activity of pacsin-2 was investigated in in vitro assays [114]. Interestingly, the presence of FlnA increased the tubulation activity and changed the average diameter of the tubules from 53 to 77 nm (Fig. 5). FlnA was not associated with the pacsin-2 coat of these tubules, thereby indicating that FlnA domain 20 promotes pacsin-2 specific membrane tubulation processes. Furthermore, FlnA was shown to be essential for the localization of pacsin-2 to specific membrane sites in platelets (presumably to the open canalicular system) and to the demarcation membrane system in midstage megakaryocytes.

### (Patho)physiological interactors of the PSTPIP1 F-BAR domain

#### Binding of a proline-rich peptide sequence of PEST PTP

The F-BAR domain protein PSTPIP1 (Proline, Serine, Threonine-rich Phosphatase Interacting Protein 1) was independently identified in two-hybrid screens as an interactor of the PEST-type protein tyrosine phosphatase (PEST PTP) [121] and as interactor of CD2 [122]. It was found to be a substrate for this phosphatase in v-Src-transfected cells. The phosphatase bound to PSTPIP1 via its C-terminal 24 amino-acid long proline-rich domain. Surprisingly, although PSTPIP1 contained a SH3 domain, the binding site of PSTPIP1 for PEST PTP was localized to the coiled-coil region, later recognized as the F-BAR domain. A tryptophan at position 232 was found to be essential for PEST PTP binding [123]. The interaction between PTP Pest and PSTPIP1/CD2BP1 plays a role in CD2-specific activation of T cells [124]. Interestingly, mutations in the F-BAR domain (E250Q and A230T) of CD2BP1/PSTPIP1 are associated with the PAPA syndrome (acronym for Pyogenic Arthritis, Pyoderma gangrenosum, and Acne), a rare inherited auto-inflammatory disease, and to disrupt the binding of PTP PEST [125].

#### Pyrin

Again by a two-hybrid screen, PSTPIP1 was found to interact with pyrin, a protein mutated in a systemic auto-inflammatory disease called familial Mediterranean fever (FMF) [126]. The B box/coiled-coil segment of pyrin and both the SH3 domain and the F-BAR domain of PSTPIP1 are necessary for this interaction. The absence of the tyrosine phosphorylation site in the PSTPIP1 Y344F mutant strongly decreases pyrin-binding in cells treated by phosphatase inhibitors, indicating that the association is dependent on tyrosine phosphorylation. Moreover, the F-BAR domain mutants E250Q and A230T associated with the PAPA syndrome showed increased pyrin-binding, suggesting that the pyrin-PSTPIP1 interaction plays a central role in both in FMF and PAPA syndrome. Waite et al. showed that PSTPIP1 dimers induced perinuclear membrane filaments dependent on an intact microtubular system [127]. PAPA syndrome mutants were not compromised in this property of filament formation. However, co-expression of pyrin recruited this protein to PSTPIP1 filaments and induced the remodeling of the filaments into a more branched and reticular network. In turn, PSTPIP1 was partially recruited to the inflammasome compartment by pyrin and this recruitment was enhanced in PAPA syndrome mutants. Moreover, Starnes et al. revealed that PSTPIP1 negatively regulates podosome formation and matrix generation in macrophages and identified a PAPA syndrome mutation within the SH3 domain that shows alterations in WASP activity and actin cytoskeleton organization [128]. Recently, a third auto-inflammatory syndrome was identified to be caused by mutations in the PSTPIP1 gene and alterations in the PSTPIP1-pyrin interaction: hyperzincemia and hypercalprotectinemia (Hz/Hc) [129]. Interestingly, the PSTPIP1 point mutations of Hz/Hc patients (E250K and E257K) are localized close to the mutations associated with the PAPA syndrome and reveal an acidic patch at the convex side of the BAR domain that is responsible for the regulated association of pyrin. The charge reversal mutant E250K in the Hz/Hc patients led to an even stronger association of pyrin as compared to the PAPA syndrome E250Q mutant. This variation in the PSTPIP1-pyrin affinity is likely the cause of the differential symptoms between these two syndromes as for example the extremely high secretion of alarmin-type cytokines in Ht/Hc.

### Interaction between CDC15 and CDC12 promotes cytokinesis

In *Schizosaccharomyces pombe*, cytokinesis and the assembly of the cytokinetic actin ring is critically dependent on the F-BAR domain protein CDC15 [130]. Assembly and maturation of the cytokinetic actin ring are assumed to involve a sequence of events starting with dephosphorylation of the hyperphosporylation state of interphase CDC15, binding of the formin CDC12 by CDC15, localization of this complex to the midplane of the cell, and interaction of the CDC15/CDC12 complex with Myo1 which activates the Arp2/3 complex for promoting the final actin cable network. Hyperphosphorylation occurs mainly at the linker region between the F-BAR domain and the C-terminal SH3 domain of CDC15 and negatively regulates the association between CDC15 and CDC12 [131]. The binding itself is conferred by the F-BAR domain of CDC15 and a short motif in the N-terminus of CDC12 (amino acids 20–40) with a dissociation constant of about 2 nM [17]. It has not been tested yet whether the CDC12-binding interferes with membrane association of the BAR domain; however, a simultaneous rather than a competitive binding is likely and would allow the tight connection between the membrane and the cytokinetic actin ring during cell division.

## Phosphorylation-dependent regulation of activity

### A phosphosite in the H_0_ helix of the endophilin N-BAR domain

Phosphorylation of endophilin A1 on T14 in the N-terminal amphipathic H_0_ helix by Rho kinase has been observed during clathrin-mediated endocytosis of the EGF receptor. Expression of a phosphomimetic mutant T14D inhibited the internalization of the EGF receptor, whereas endophilin wild-type or a T14A mutant did not [132]. In an early step of endocytosis, endophilin is recruited via CIN85 and Cbl to form a complex with activated EGF receptors. CIN85 binding and complex formation is likely impaired by the T14 phosphorylation. Thus, this phosphorylation seems to regulate the recruitment rather than the membrane-binding properties of endophilin. Considering that the H_0_ helix is also a site of auto-regulation [87], the T14 phosphorylation may also affect the inhibitory effect of the SH3 domain of the second subunit of the dimer. Hence, further studies are necessary to assess the effect of this phosphosite on auto-inhibition and membrane binding to fully elucidate the regulatory mechanism of this endophilin phosphorylation in receptor endocytosis.

### A phosphosite in the endophilin-specific central insert region

**Fig. 6:**
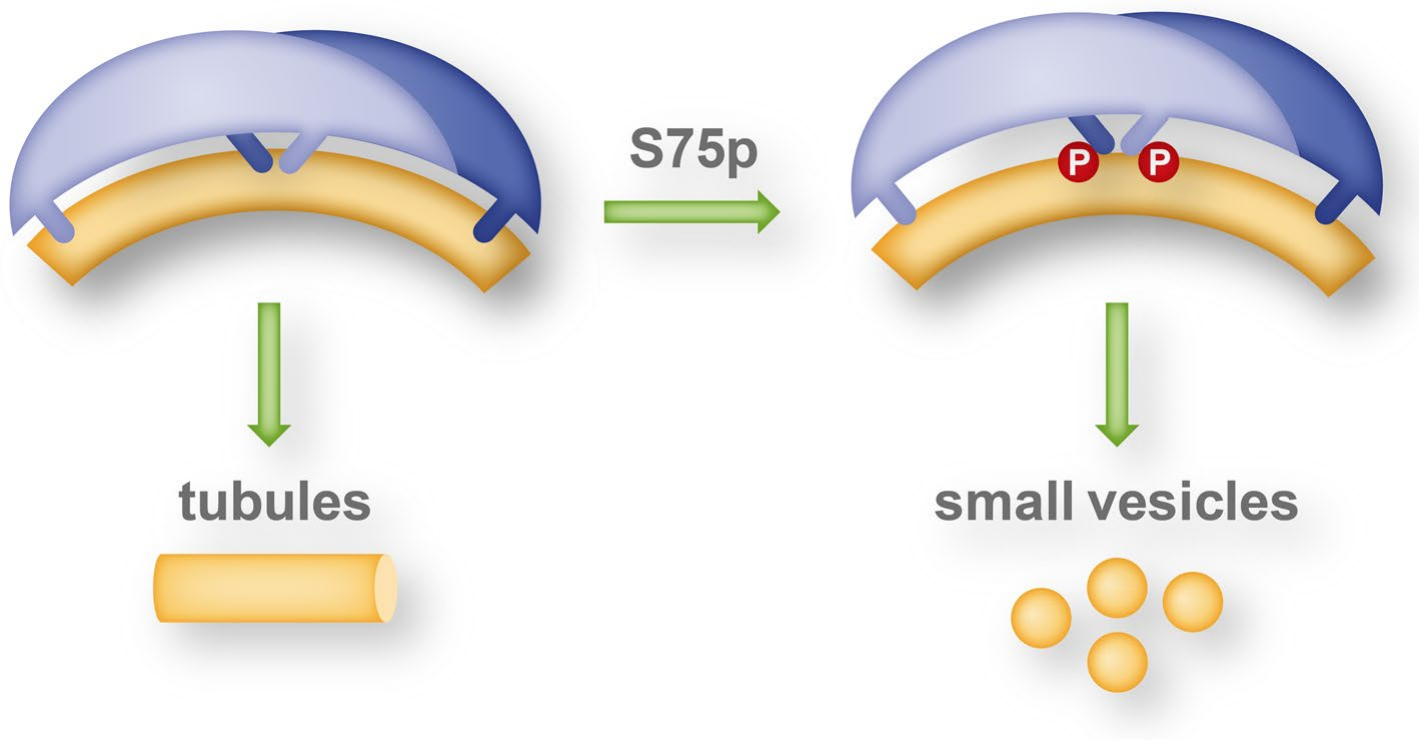
Phosphorylation of the central insert of endophilin A1 controls its generation of membrane shapes. Apart from its N-terminal amphipathic helices at the tips of the BAR-domain dimer, endophilin A1 (*dark* and *light blue moons*) contains an additional pair of amphipathic helices (also termed central insert region). Shallow insertion of the amphipathic helices preferentially stabilizes small vesicles, whereas deep insert of these helices and tight contact of the BAR-domain with the headgroups of the membrane phospholipids favor membrane tubulation. Phosphorylation of the central insert region at S75 (*red circles*) by the Parkinson disease-associated kinase LRKK2 controls the membrane insertion depth of the amphipathic helices and thereby the type of membrane curvature generated by endophilin A1 [58]

A further phosphosites of endophilin A1, S75, plays an important role in clathrin-mediated endocytosis of synaptic vesicles and subsequent neurotransmission at the synapse in *Drosophila* [133]. S75 is located within the endophilin-specific appendage that constitutes the central insert region of the BAR-domain dimer and is phosphorylated by LRRK2, a kinase that is mutated in Parkinson’s disease. Endophilin phosphorylated by LRRK2 has reduced membrane affinity and tubulating activity in vitro and in vivo. Interestingly, an EPR study by Ambroso et al. revealed that the phosphomimetic S75D mutant lost the ability of deeply inserting the central insert region into the membrane bilayer and was predominantly found associated with small vesicles rather than tubules in vitro [58] (Fig. 6). LRRK2-specific phosphorylation of S75 and to a lesser extend of T73 was recently also shown to affect mammalian synaptic function and it is assumed that a carefully balanced regulatory system of this endophilin A1 phosphorylation site is required for a proper functioning of the synaptic vesicle cycle [134].

### Two phosphosites at helix-capping motifs in the F-BAR domain

Pacsin 1 (syndapin I) interacts with dynamin 1 in nerve terminals and is thereby involved in activity-dependent but clathrin-independent bulk endocytosis [135, 136]. It moreover functions in neuronal morphogenesis being involved in neurite outgrowth and branching [28]. Quan et al. identified two phosphosites in the F-BAR domain of pacsin 1, S76 and T181, which affect the membrane-binding property and tubulating activity of this protein [137] (Fig. 5). Both residues are evolutionary conserved and are located at helix-capping motifs, S76 at helix H_2_ in the central 6 helix bundle region of the F-BAR domain dimer, and T181 at helix H_4_ at the distal tip of the dimer. These residues are also conserved and located at the same structural position in other F-BAR proteins, Cip4, FBP17, and FCHO2, suggesting a more general importance of these phosphosites for the regulation of F-BAR domain activity. Modeling analyses suggest that these phosphosites affect the F-BAR function by different mechanisms: S76 phosphorylation may alter the curvature of the F-BAR dimer, whereas T181 phosphorylation may interfere with tip-to-tip inter-dimer association necessary for filament assembly. In fact, both phosphomimetic mutants showed altered liposome-binding and tubulation activity in vitro, however, with different specificities. Intracellular tubulation upon over-expression was absent in the T181E mutant and significantly reduced in S76E mutants as compared to wild-type pacsin 1. The phosphomimetic mutants did not affect bulk endocytosis, but especially T181E was found to be developmentally regulated and did affect neuromorphogenesis and neurite outgrowth. The kinase and signaling pathway that are responsible for these phosphorylations have yet to be explored and this will be essential to fully evaluate the cell biological importance of this posttranslational modification.

### Phosphosites in arfaptins

Gehart et al. identified an essential step in the regulatory mechanism of secretory granule biogenesis at the trans-Golgi network (TGN), a process which is thought to be critically impaired in pancreatic β cells of type II diabetic patients [138]. They found that the BAR domain protein arfaptin-1 stabilizes the narrow neck of a budding granule precursor and thereby prevents the scission of immature insulin granules at the TGN. Interestingly, rather than binding to the highly curved membrane neck alone, arfaptin-1 dimers likely bind both the membrane and membrane-bound active ARF involving different interaction surfaces of the protein. The ARF-arfaptin coat physically stabilizes this sensitive membrane region. Furthermore, the interaction with arfaptin-1 on one hand shields ARF from associating with components of the scission complex and on the other hand likely prevents membrane fission by the amphipathic helices of ARF dimers. Final fission of mature granules is initiated by a PKD-dependent phosphorylation of arfaptin at serine 132 which is close to the N-terminus of BAR domain and has been shown to be essential for ARF binding [139]. Phosphorylation of arfaptin apparently destabilizes the interaction leading to the dissociation of arfaptin from the vesicle neck and thus promoting the process of granule fission.

An Akt-dependent phosphorylation of S260 of arfaptin-2 was shown to inhibit toxicity induced by polyQ-huntingtin indicating a neuroprotective effect of this posttranslational modification within the BAR domain. In line with this is the finding that arfaptin-2 is upregulated in Huntinton disease patients. While the involved molecular mechanism is still unclear, a rescue of impaired proteasome activity was found as a downstream effect of this phosphorylation event [140].

## Concluding remark

During the last decade, it became clear that BAR domain proteins are key players in membrane shaping processes. Each member of the BAR domain superfamily—more than 70 are by now characterized only in humans—appears to be involved in the formation of certain subcellular membrane structures. This is achieved by its specific BAR domain architecture mostly in combination with a unique set of additional domains with enzymatic, signaling, protein, or lipid-binding properties. These domains are involved in the regulated assembly of macromolecular membrane complexes and modulate the membrane sculpting activity of the BAR domain dimer. Some membrane processes involve several BAR domain proteins in a concerted manner and/or in a consecutive order. The molecular mechanisms that coordinate such processes in space and time are only slowly emerging. Innovative methods like BioID, a biotin ligase-based tagging proteomics to identify proximal partners of BAR domain proteins (at the membrane), or CRISPR/Cas9 applications that knockout/modify not only single but several BAR domains at the same time will help to unravel the molecular network behind cellular membrane morphogenesis. These efforts will also shed light on aberrations of these processes in pathological situations and will help to understand the involvement of BAR domain proteins in various disease-related disorders.
